# A Rare Case of Extramedullary Plasmacytoma Presenting as Massive Upper Gastrointestinal Bleeding

**DOI:** 10.7759/cureus.3993

**Published:** 2019-01-31

**Authors:** Isin Y Comba, Nancy E Torres Luna, Christopher Cooper, Maria W. Crespo, Allison Carilli

**Affiliations:** 1 Internal Medicine, University of Central Florida College of Medicine, Orlando, USA; 2 Pathology, Osceola Regional Medical Center, Kissimmee, USA; 3 Oncology, University of Central Florida College of Medicine, Orlando, USA

**Keywords:** multiple myeloma, plasmacytoma, extramedullary myeloma, massive upper gastrointestinal (gi) bleed

## Abstract

Gastrointestinal (GI) involvement by multiple myeloma is a rare entity. Clinical manifestations depend on the site and extent of involvement. GI bleeding, obstruction, and perforation can complicate the disease course. We report a rare case of an extramedullary plasmacytoma ulcerating through the gastric mucosa and presenting as a massive upper GI bleed, which was controlled surgically with en-bloc resection of the mass infiltrating the stomach, distal pancreas, and spleen. To our knowledge, this is the first case of immunoglobulin A (IgA) myeloma with multiple GI organ involvement presenting with massive upper GI bleeding.

## Introduction

Multiple myeloma (MM) is a plasma cell malignancy that is associated with an intramedullary proliferation of monoclonal plasma cells [1]. The recent therapeutic advances in MM management with the use of antiangiogenic thalidomide and proteasome-inhibitor bortezomib improved the overall survival of these patients [1-2]. Although most of the MM patients have only intramedullary involvement, a recent increase in the incidence of extramedullary myeloma (EMM) has been reported, possibly due to a longer lifespan with the novel regimens [3]. Gastrointestinal (GI) system involvement by MM is still a rare entity, accounting for only < 1% of MM cases [4]. The majority of the patients are diagnosed with GI involvement during follow-up visits or relapses of the MM rather than the initial diagnosis [4-5]. It portends a higher risk of relapse, poor response to conventional treatment, and overall lower survival compared with marrow-restricted myeloma [4-6]. We report a case of an aggressive extramedullary myeloma invading the stomach, distal pancreas, and spleen. Our case presented with persistent, massive upper GI bleeding which was controlled surgically with en-bloc resection.

## Case presentation

A 63-year-old male presented to the emergency department with a one-day history of melanotic stools. He also reported shortness of breath and epigastric abdominal pain. The patient denied using any non-steroidal anti-inflammatory drugs (NSAIDs) and has a remote history of alcohol abuse. He was not on anticoagulation.

The patient has a history of an immunoglobulin A (IgA)-Kappa type, solitary chest plasmacytoma treated with radiotherapy with a subsequent initial remission two years ago. Later on, another plasmacytoma in the right femoral shaft was found and treated with radiotherapy. One month before the presentation, he was diagnosed with oligosecretory MM. He was started on cyclophosphamide, bortezomib, and dexamethasone and received two cycles. On physical examination, vital signs were significant for tachycardia with a pulse of 104 beats per minute, blood pressure of 107/70 mmHg, respiratory rate of 18 per minute, and temperature of 97.5 degrees F. He appeared in mild respiratory distress and was noted to be pale. Bowel sounds were present, and the abdomen was soft, non-tender, and non-distended.

Laboratory tests on admission showed a hemoglobin of 6.5 g/dL (normal range: 13 - 17), a white blood cell (WBC) count of 4.5 k/mm3 (4.2 - 10.3), and a platelet count of 121 k/mm^3^ (150 - 410). After a one unit packed red blood cell (RBC) transfusion, his hemoglobin came back 5.4 g/dL. Additionally, his other laboratory studies showed a prothrombin time (PT) of 14.6 sec, internationalized normalized ration (INR) of 1.29, urea nitrogen of 27 mg/dL (7 - 20.6), creatinine of 1.1 mg/dL (0.7 - 1.3), calcium of 8.6 mg/dL (8.4 - 10.6), total protein of 6.5 g/dL (6.4 - 8.3), albumin of 2.6 g/dL (2.8 - 4.5), and a lactate dehydrogenase (LDH) of 229 U/L (125 - 220).

His last positron emission tomography-computed tomography (PET-CT) scan revealed hypermetabolic lesions in the right kidney, stomach, spleen, pancreas, and right proximal femur. His last immunofixation study demonstrated an immunoglobulin M (IgM) level of 23 mg/dL (40 - 230), immunoglobulin G (IgG) of 373 mg/dL (700 - 1,600), IgA of 502 mg/dL (91 - 414), and kappa/lambda ratio of 6.59 (0.28 - 1.65).

**Figure 1:**
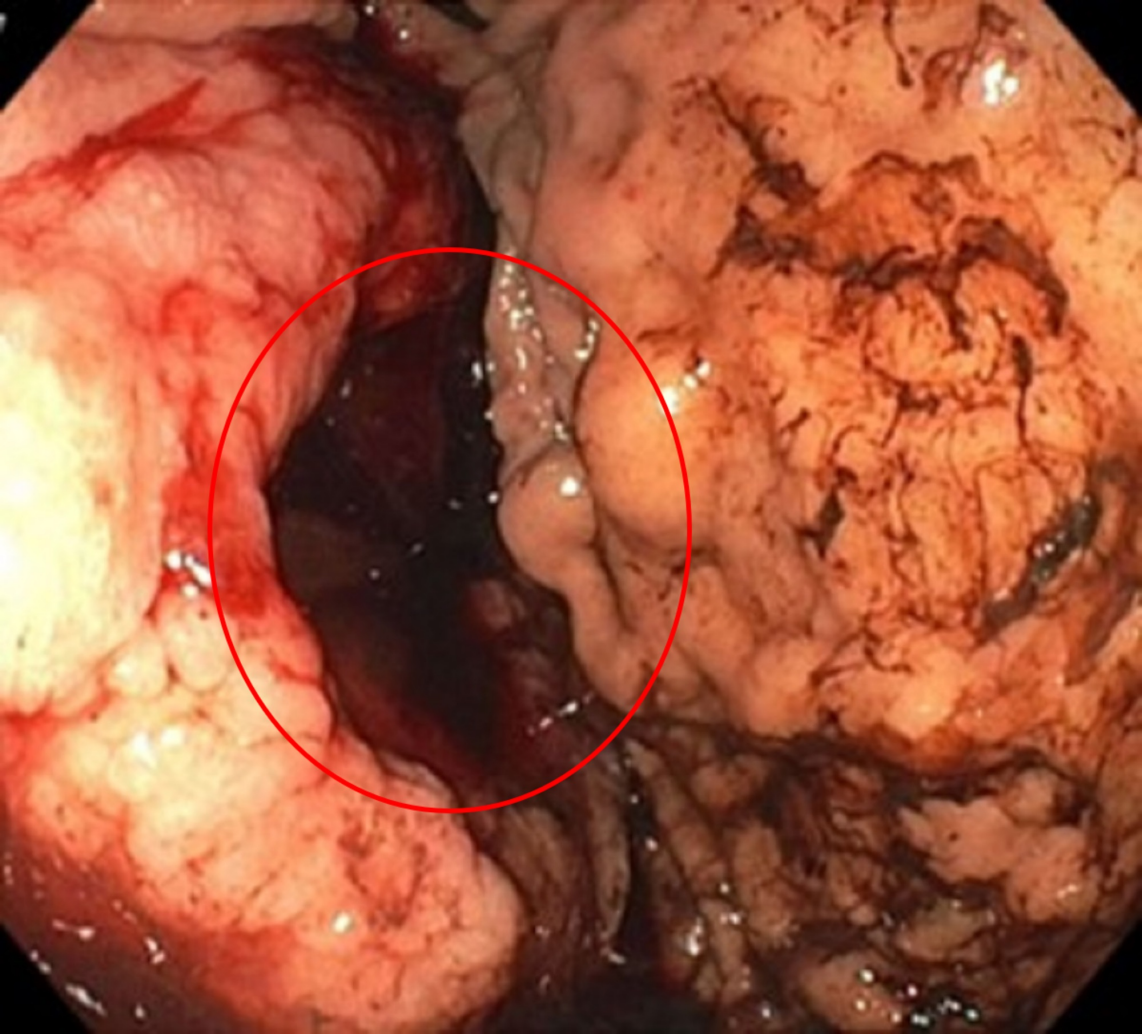
Endoscopic image Endoscopic image demonstrating an infiltrative mass which ulcerated along the greater curvature starting from proximal body.

**Figure 2:**
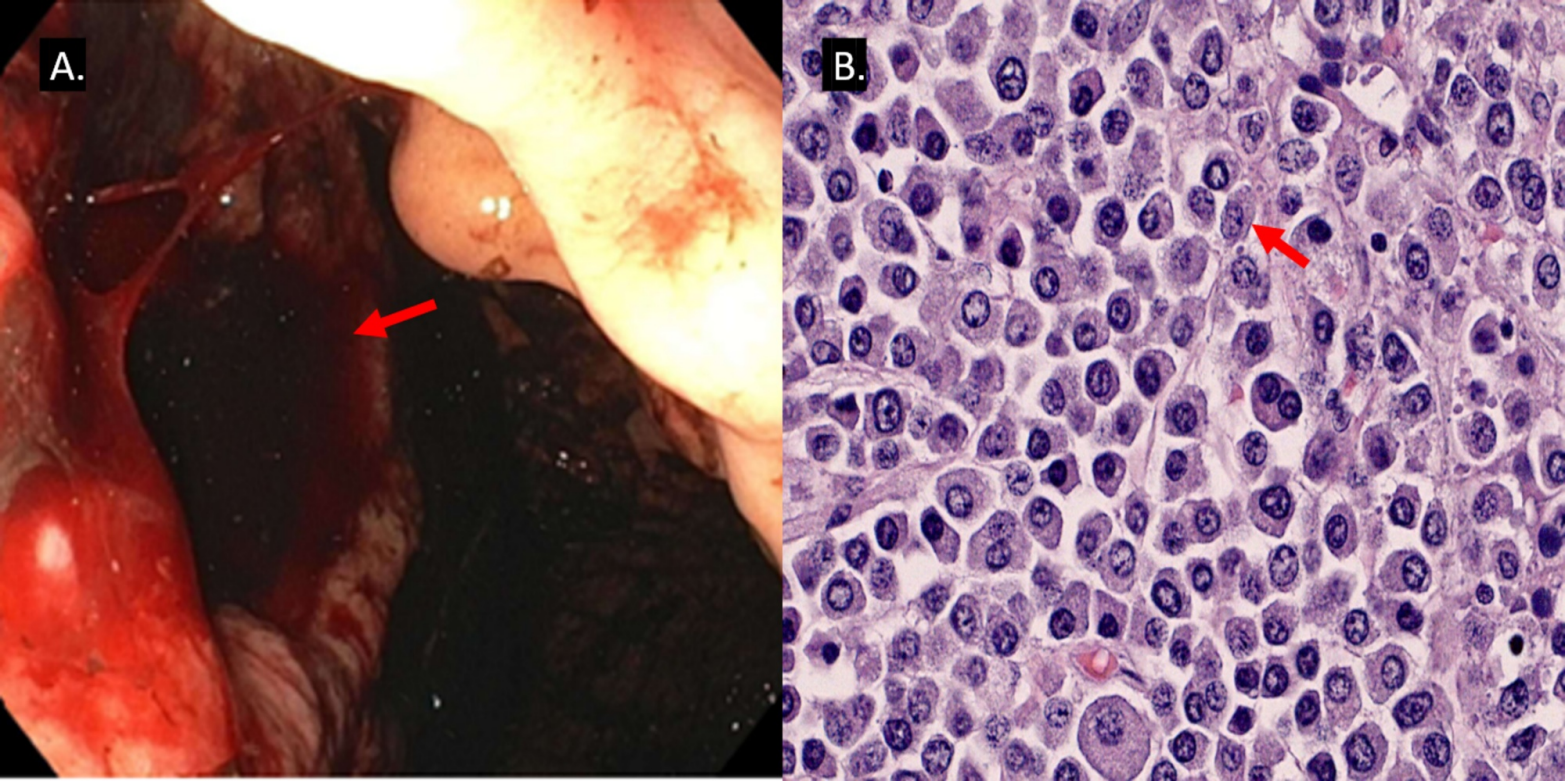
Endoscopic image (A) and histological section (B) A) Endoscopic image showing a large (> 7 cm), actively bleeding ulcer in the gastric cardia; B) Histological section of the surgical sample demonstrating atypical cells showing enlarged eccentric nuclei with prominent nucleoli, eccentric cytoplasm, and moderate basophilic cytoplasm.

After initial fluid resuscitation and blood transfusions, he had an emergent esophagogastroduodenoscopy which showed a deep, cratered, oozing gastric ulcer measuring at least > 7 cm on the proximal body extending posteriorly to the greater curvature of the gastric body with adherent clots (Figures 1-2A). The patient underwent a subsequent embolization by interventional radiology of the short gastric and left gastric arteries. Over the next 72 hours, he continued to have persistent, severe bleeding requiring transfusion of 8 units of packed red blood cells (PRBCs). Emergent explorative laparotomy was done and revealed a large 9 x 9 x 7 cm ulcerating mass extending through the mucosa of the stomach with invasion into the surrounding soft tissues. The mass involved the adipose tissue around the stomach, the splenic, and pancreatic parenchyma and surrounded the splenic vein. A 4 cm liver mass in the right lobe was also noted. En-bloc resection of the greater curvature of the stomach, spleen, and distal pancreas was done with successful control of the bleeding. Histopathologic evaluation of the surgical sample demonstrated a high-grade (anaplastic plasma cell) plasmacytic neoplasm (cd138+, cd79a+, mum1+, negative for CD45, CD20, CD56, CD43, PAX5, BLC2, Eber, and HHV-8) involving the greater curvature of the stomach, distal pancreas, and spleen (Figure 2B). Ki-67 demonstrated 30% nuclear staining.

The patient remained symptom-free with no evidence of GI bleeding after the surgery. He was discharged from the hospital at postoperative day 9 for follow-up with his oncologist. He was started on high-dose chemotherapy with plans for a subsequent bone marrow transplant (BMT).

## Discussion

The existence of myeloma cells at distant sites from the bone marrow and surrounding soft tissues in MM patients is termed extramedullary myeloma (EMM) [6-7]. The incidence of EMM is 6% - 8% at initial MM diagnosis and up to 30% over the course of the disease [7]. A higher incidence of TP53, RB1 mutations, focal adhesion kinase (FAK), and renin-angiotensin system (RAS) oncogene activation, increase in CD44, and loss of CD56 cell surface antigen are seen in EMM compared with marrow-localized myeloma [4, 6]. Additionally, an increase in angiogenetic factors and reduction in cell adhesion molecules have been shown in some studies [6-8]. Despite these findings pointing out the distinct features of EMM, the pathogenesis of this disease process remains largely unknown [6].

The GI tract is rarely involved in multiple myeloma [4-5]. In a systemic review done with 2,584 MM patients, the prevalence of GI system involvement was 0.9%. The prognosis of these patients was poor with median survival less than seven months from diagnosis [4]. Clinical manifestations of EMM infiltrating the GI system vary depending upon the site, extent, and duration of involvement. Patients can be asymptomatic, have non-specific GI symptoms, or present with potentially life-threatening complications, including GI hemorrhage, obstruction, and perforation [4, 9]. GI bleeding as a complication of stomach and/or bowel involvement is reported in a few case reports [9-14]. To our knowledge, this is the first case of IgA myeloma with multiple GI organ involvement, including the stomach and pancreas. In contrast to other reported cases, the severity of the GI bleeding warranted surgical control.

EMM is identified as high-risk myeloma [15]. It has higher mortality and poor response to conventional treatments compared with marrow-localized myeloma [15]. Positron emission tomography-computed tomography (PET-CT) is essential in the detection of the extramedullary lesions [6-7]. The individual clinical and biological features should be taken into consideration in establishing the treatment protocol, as well as each eligible patient being evaluated for the bone marrow transplant [15].

GI light chain (AL) amyloidosis needs to be considered in the differential diagnosis as it has been reported to cause gross GI hemorrhage in MM patients as a result of amyloid fibril deposition in the vessel walls of the GI tract. Histopathologic sampling is essential for the diagnosis of GI amyloidosis [16-18].

It is important to note that abnormalities in primary and/or secondary hemostasis due to paraprotein-induced platelet-function and coagulation factors impairment, chemotherapy-induced or underproduction thrombocytopenia, concurrent use of anticoagulation prophylactically with thalidomide or lenalidomide, or acquired Von Willebrand factor deficiency can increase the risk of GI bleeding in MM patients [19].

## Conclusions

In this report, we describe an aggressive extramedullary myeloma case with the involvement of multiple GI organs that presented with massive GI bleeding and was treated with surgical en-bloc resection. Taken together, the gastrointestinal tract is an infrequent site of involvement in patients with MM. Patients with GI invasion of myeloma can present with indolent or overt GI bleeding. Additionally, bowel perforation or obstruction can complicate the disease course. Extramedullary myeloma is classified as high-risk myeloma with poor response to conventional myeloma therapies. In determining the treatment protocol, the biological characteristics of the disease and individual clinical features are key. Particularly, each eligible patient should be evaluated for the bone marrow transplant early in the course.
